# Supplementary material for: Distinct neurochemical influences on fMRI response polarity in the striatum
Source: Nat Commun. 2024 Mar 1;15:1916. doi: 10.1038/s41467-024-46088-z (PMC10907631; doi:10.1038/s41467-024-46088-z)
Supplement: Supplementary file 1 — Supplementary Information [file 41467_2024_46088_MOESM1_ESM.pdf]

Distinct neurochemical influences on fMRI response polarity in the striatum

Supplementary Information

I. Supplementary Figures -- Page 2

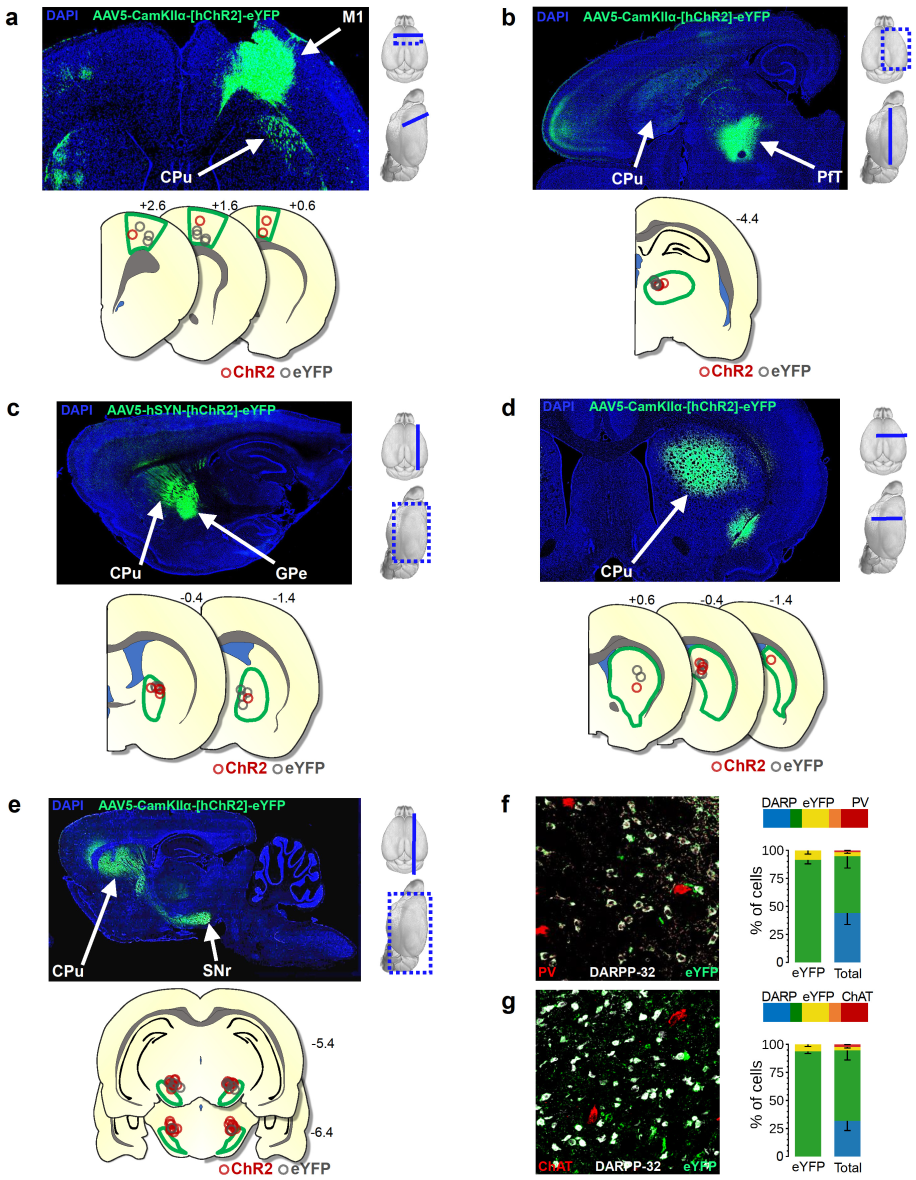

Fig. S1: Confirmation of optogenetic fMRI stimulation targets in Figure 1. a-e (top) Representative cross-section indicating spread of eYFP for each stimulation target, with 3-D models of the brain to the right, indicating the location of the histological slice. **a-e** (bottom) Locations of optical fiber tips relative to the anatomical stimulation target ROI outlined in green. Stimulation targets and areas of interest are as follows: **a** Viral expression in M1 injection sites and its spread to CPu. **b** Viral expression at the PfT injection site and its spread to CPu. **c** Viral expression at the GPe injection site and its spread to CPu. **d** Viral expression in the CPu for direct MSN stimulation. **e** Viral expression at the CPu injection site and its spread to SNr stimulation sites. **f-g** High-resolution 20x magnification representative image and immunohistochemical verification of DARPP-32 (DARP), eYFP,

and either PV (**f**) or ChAT (**g**) co-expression in striatum (PV n = 2 brains, 7 slices, 6195 cells; ChAT n = 3 brains, 11 slices, 14181 cells; data are presented as mean ±SEM). Source data are provided as a Source Data file.

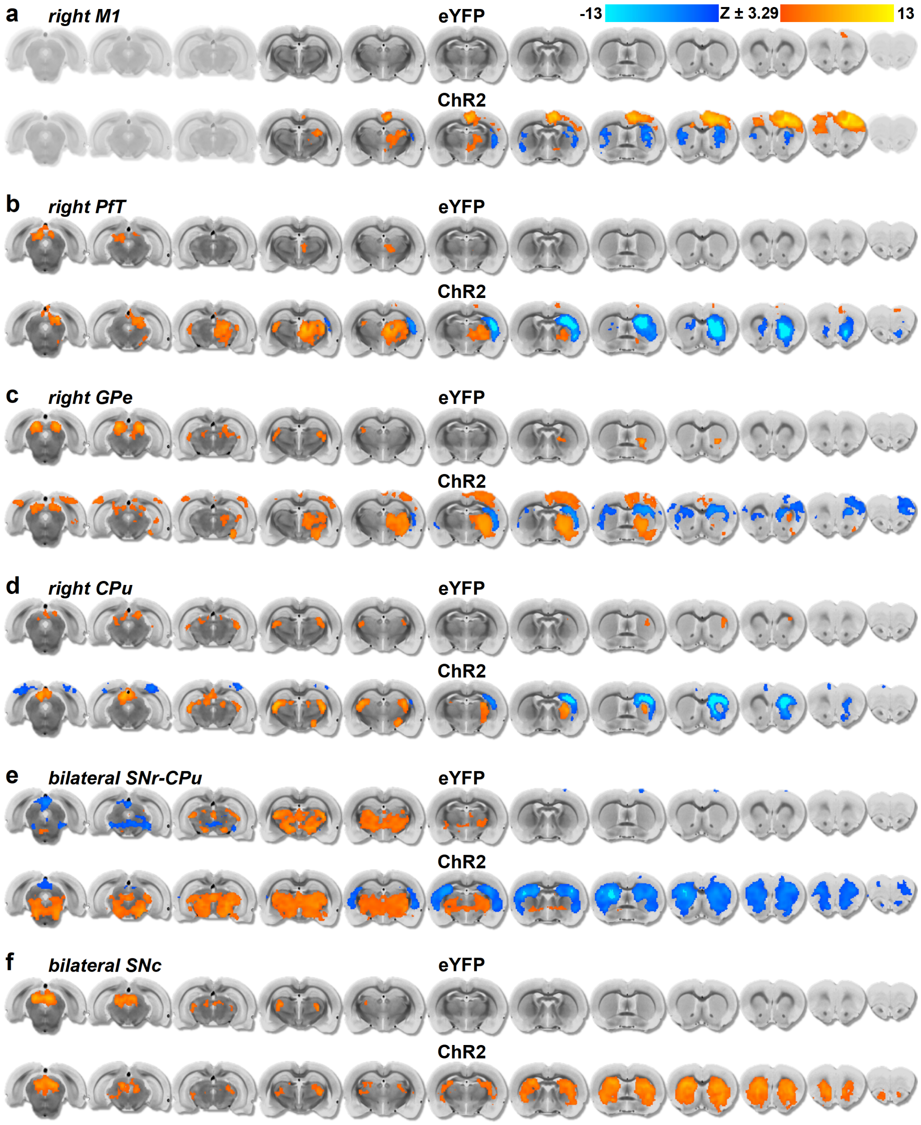

Fig. S2: Extended 12-slice optogenetic CBV-fMRI response maps acquired during CPu circuit manipulations in Figure 1. a-f Response maps acquired from eYFP control and ChR2 subjects following optogenetic stimulation of the circuit indicated at the top left of each set of maps**.** Left-to-right corresponds to 1 mm steps in the posterior-to-anterior direction, with the 5th slice from the right located at the anterior commissure (approximately –0.36 mm AP). Response maps thresholded to $p < 0.001$

(two-tailed), FWE corrected to $\alpha < 0.01$. Note that responses in visual-related areas are likely the result of activity induced by side effects of photostimulation.

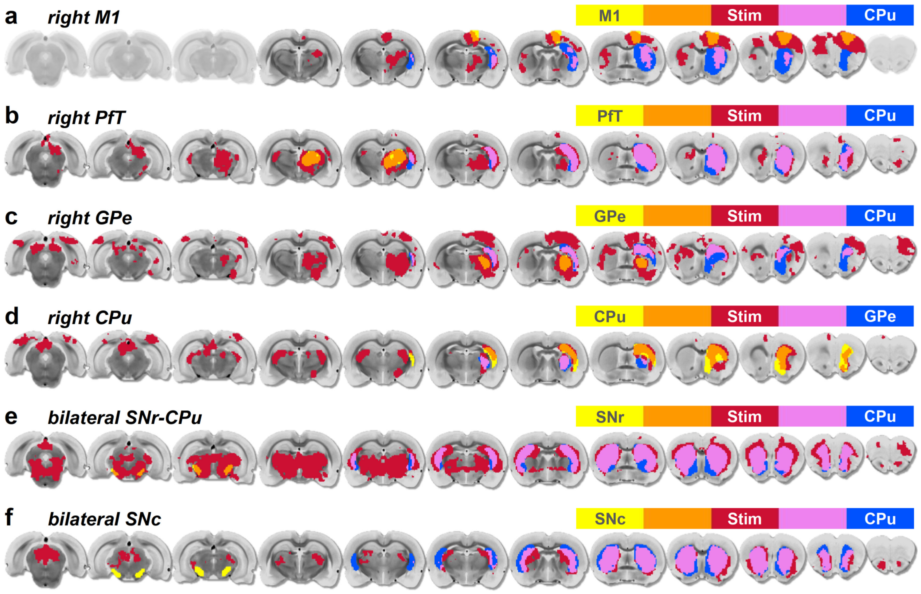

Fig. S3: Extended 12-slice optogenetic fMRI ROI maps from CPu circuit manipulations in Figure 1. a-f ROIs used for timeseries extraction were collected from the intersection of the stimulus evoked response maps (red) and anatomical stimulation targets (yellow) or downstream anatomical areas of interest (blue), corresponding to the orange and purple regions, respectively. **f** Note, no stimulus evoked response was detected within the anatomical boundaries of SNc for bilateral SNc dopamine neuron stimulation. Left-to-right corresponds to 1 mm steps in the posterior-to-anterior direction, with the 5th slice from the right located at the anterior commissure (approximately –0.36 mm AP).

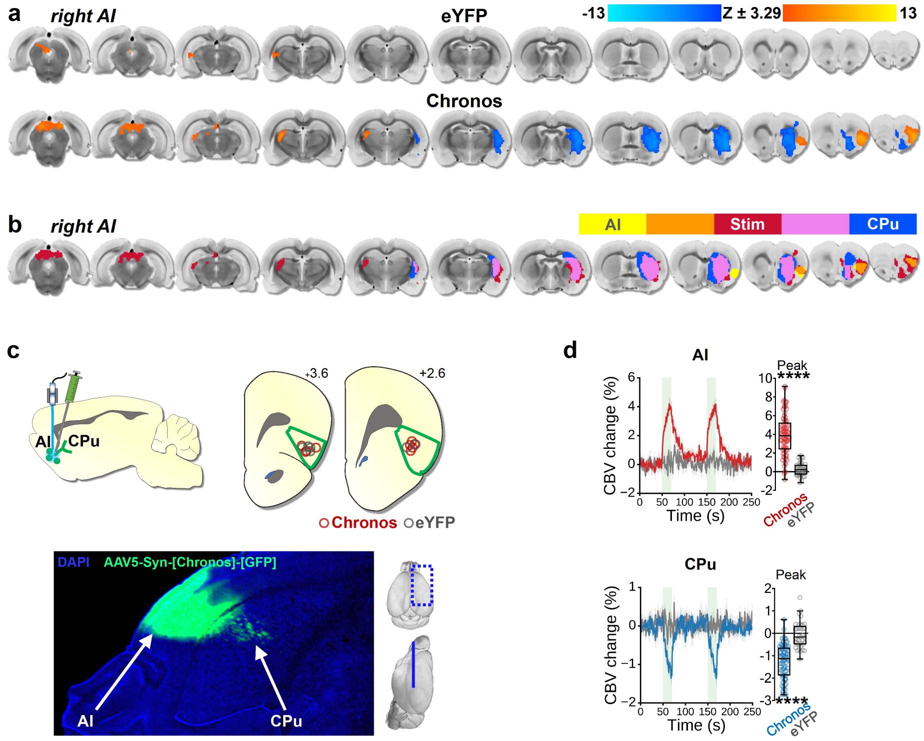

Fig. S4: 20 Hz optogenetic stimulation of anterior insular cortex (AI) during CBV fMRI. a fMRI response maps acquired from eYFP control and Chronos subjects following optogenetic stimulation of the right AI**.** Response maps thresholded to $p < 0.001$ (two-tailed), FWE corrected to $\alpha < 0.01$. **b** ROIs used for timeseries extraction were collected from the intersection of the stimulus evoked response map (red) and anatomical boundary of AI (yellow) or CPu (blue), corresponding to the orange and purple regions, respectively. **a-b** Left-to-right corresponds to 1 mm steps in the posterior-to-anterior direction, with the 5th slice from the right located at the anterior commissure (approximately –0.36 mm AP). **c** Stimulation schematics (top left), where AI viral expression and projections to CPu are indicated in green (not all projections shown) and optogenetically stimulated area of AI is indicated in blue; locations of optical fiber tips for AI optogenetic stimulation (top right); representative tissue cross-section indicating spread of eYFP for from AI injection site (bottom), with 3-D models of the brain to the right indicating the location of the histological slice. **d** CBV time-courses from AI response maps (left) and CPu response maps (right) aligned to stimulation epochs (green bars indicate 20 Hz optogenetic stimulation blocks; data are presented as mean ±SEM), with corresponding quantified peak amplitude changes (Chronos vs. eYFP two-tailed Welch's t-test, ****$p < 0.0001$; box plots span IQR, with median line and whiskers within bounds ±1.5 IQR, using Tukey's method.). **a-d** AI stimulation (20 s on, 80 s rest; 20 Hz, 10 mW power at fiber tip, 5 ms pulse-width) during CBV fMRI data (Chronos n = 9 rats, 40 epochs, 80

peaks; eYFP n = 6 rats, 18 epochs, 36 peaks). Exact p-values and test statistics are in Source Data. Source data are provided as a Source Data file.

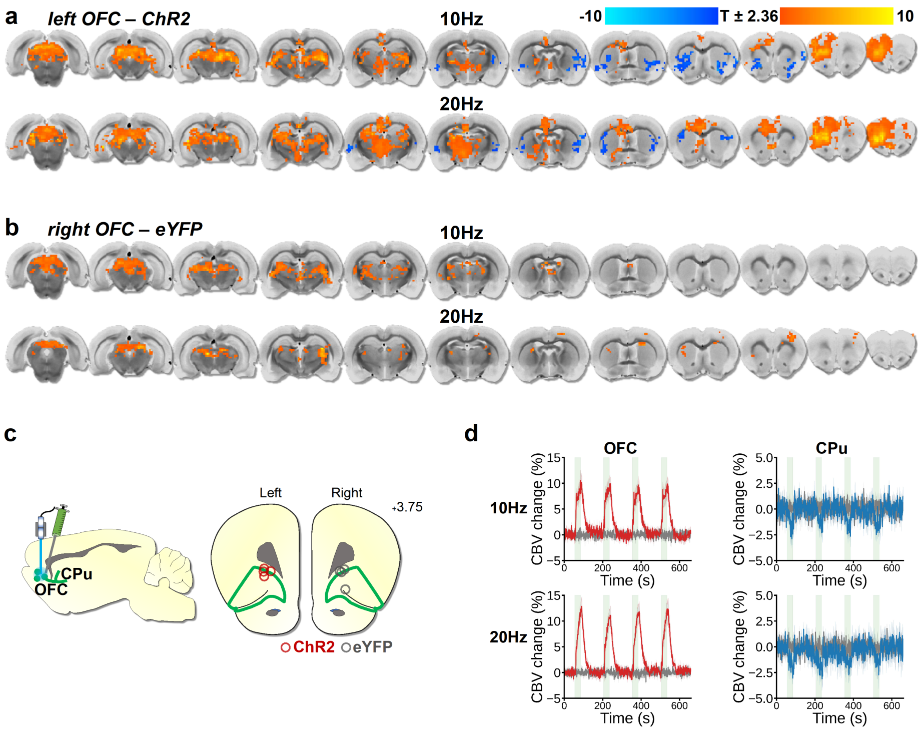

Fig. S5: 10 and 20 Hz optogenetic stimulation of orbitofrontal cortex (OFC) during CBV fMRI. fMRI response maps were acquired from unilateral optogenetic stimulation of the OFC (5 ms pulse width, 473 nm wavelength, 10 mW power), using a paradigm of 30 s stimulation off, followed by 15 s stimulation on then 60 s off repeated a total of four times, in either the ChR2-containing (left) hemisphere, **a**, at 10 Hz (top) and 20 Hz (bottom), or the eYFP-containing control (right) hemisphere, **b**, at 10 Hz (top) and 20 Hz (bottom), of four rats (3 male, 1 female). **a-b** Response maps were obtained from one-sample two-tailed t-tests using the AFNI[4] 3dttest++ command and thresholded to $p < 0.05$ without multiple comparison correction due to the limited statistical power of this proof-of-concept experiment. Instead, a cluster voxel size > 40 was applied. Left-to-right images correspond to 1 mm steps in the posterior-to-anterior direction, with the 5th slice from the right located at the anterior commissure (approximately –0.36 mm AP). Before group-level analysis, data were preprocessed for slice timing and motion corrections with AFNI and spatially aligned to the Tohoku rat brain template[1] using ITK-SNAP.[2] Functional images were smoothed with a 0.5 mm FWHM Gaussian kernel and first-level GLM was conducted via AFNI 3dDeconvolve against the MION hemodynamic response function convolved with the stimulation paradigm, which also included a second-order polynomial curve and motion parameters. **c** Stimulation schematics (left), where OFC viral

expression and projections to CPu are indicated in green (not all projections shown) and optogenetically stimulated area of OFC is indicated in blue; locations of optical fiber tips for OFC optogenetic stimulation (right), implanted 0.5 mm above the viral infusion site. **d** CBV time-course data for 10 Hz stimulation (top) and 20 Hz stimulation (bottom) was extracted from experimenter defined ROIs corresponding to the generated evoked contrast maps, trimmed to the anatomical boundaries of the OFC (left) and CPu (right) regions (green bars indicate optogenetic stimulation blocks; data are presented as mean ±SEM; n = 4 rats). All other surgical procedures, animal preparation for scanning and scanning protocol, and the percent CBV change for time-course data calculation was as described in the main Methods.

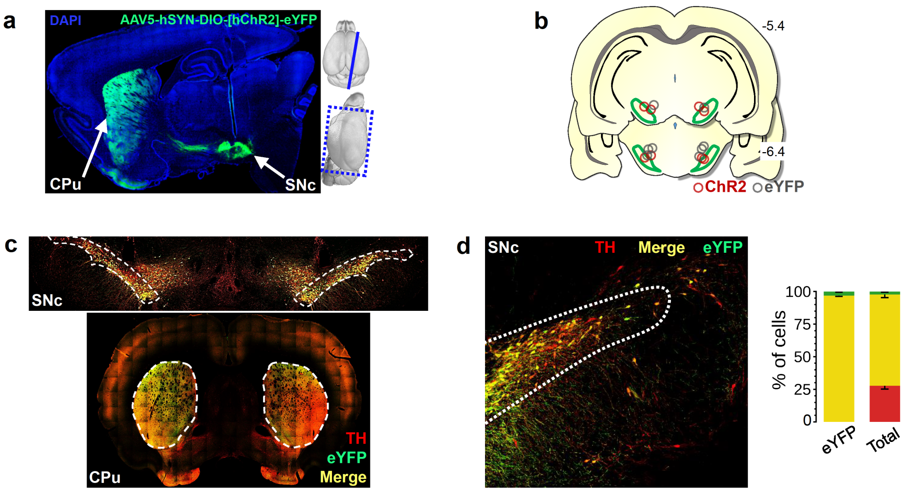

Fig. S6: Confirmation of optogenetic fMRI SNc dopamine neuron stimulation targets in Figure 1. a Representative sagittal cross-section indicating spread of TH-Cre-selective ChR2-eYFP expression from the injection site in SNc to the projection site in CPu. 3-D models of the brain are to the right, indicating the location of the histological slice. **b** Locations of optical fiber tips for SNc optogenetic stimulation with fMRI. Anatomical SNc ROI is outlined in green. **c** Representative image for high-resolution immunohistochemical verification of bilateral TH and eYFP coexpression in SNc and striatum. **d** Immunohistochemical staining used to verify expression of eYFP fluorophore in TH-expressing cells in the SNc (n = 3 brains, 22 slices, 5911 cells); percentages calculated relative to eYFP-positive cells only (left column) or all cells (right column) in SNc target area (data are presented as mean ±SEM). Source data are provided as a Source Data file.

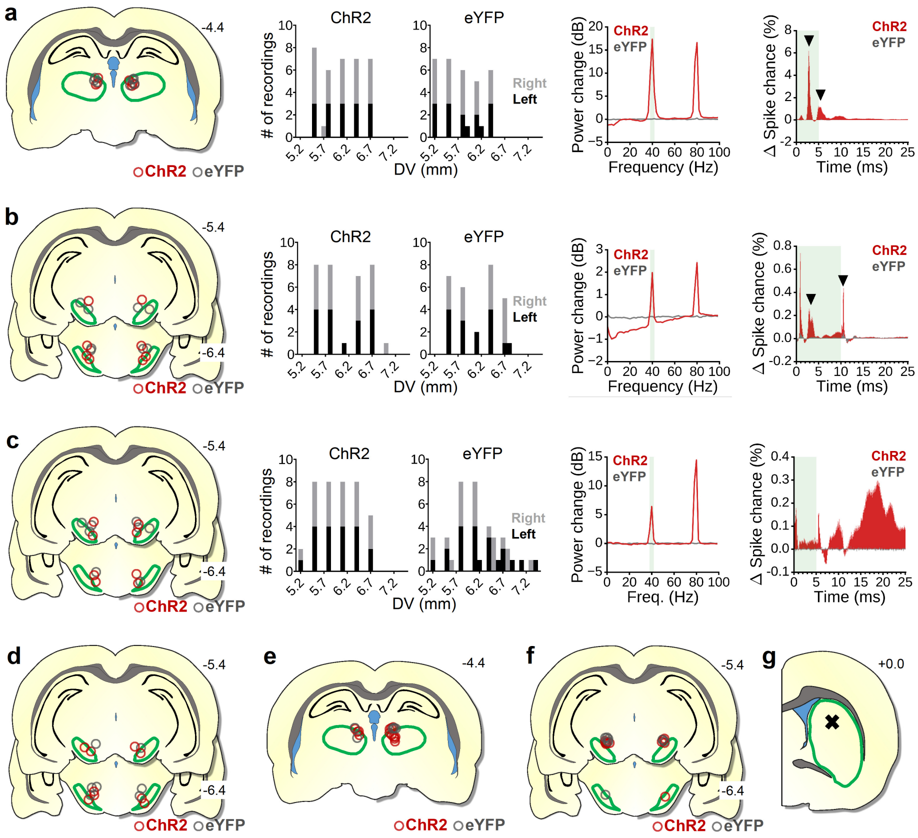

Fig. S7: Verification of optogenetic stimulation targets, electrode placements, and electrophysiology and FSCV recordings in Figure 2. a-c Left to right columns: Locations of optical fiber tips relative to the anatomical stimulation target ROI (outlined in green) for electrophysiology experiments; Acute electrophysiology electrode array implantation depths, where color indicates the recorded hemisphere; Resulting LFP power spectrum change relative to baseline from 40 Hz (highlighted in green) stimulations (data are presented as mean ±SEM); Change in average multi-unit spike probability for the period following each stimulation pulse (green shaded area) compared to baseline (data are presented as mean ±SEM). **a** 10 s, 40 Hz, 5 ms pulse-width PfT stimulation. **b** 60 s, 40 Hz, 10 ms pulse-width SNr stimulation. **a, b** (right column) Black down arrows (left to right) highlight prominent peaks at ~3 ms and at offset of ChR2 stimulation. **c** 10 s, 40 Hz, 5 ms pulse-width SNc dopamine neuron stimulation. **d-f** From left to right: Locations of optical fiber tips for SNc, PfT, and SNr optogenetic stimulation with FSCV, respectively. **g** Acute FSCV electrode implantation depth for both ChR2 and eYFP subjects. Source data are provided as a Source Data file.

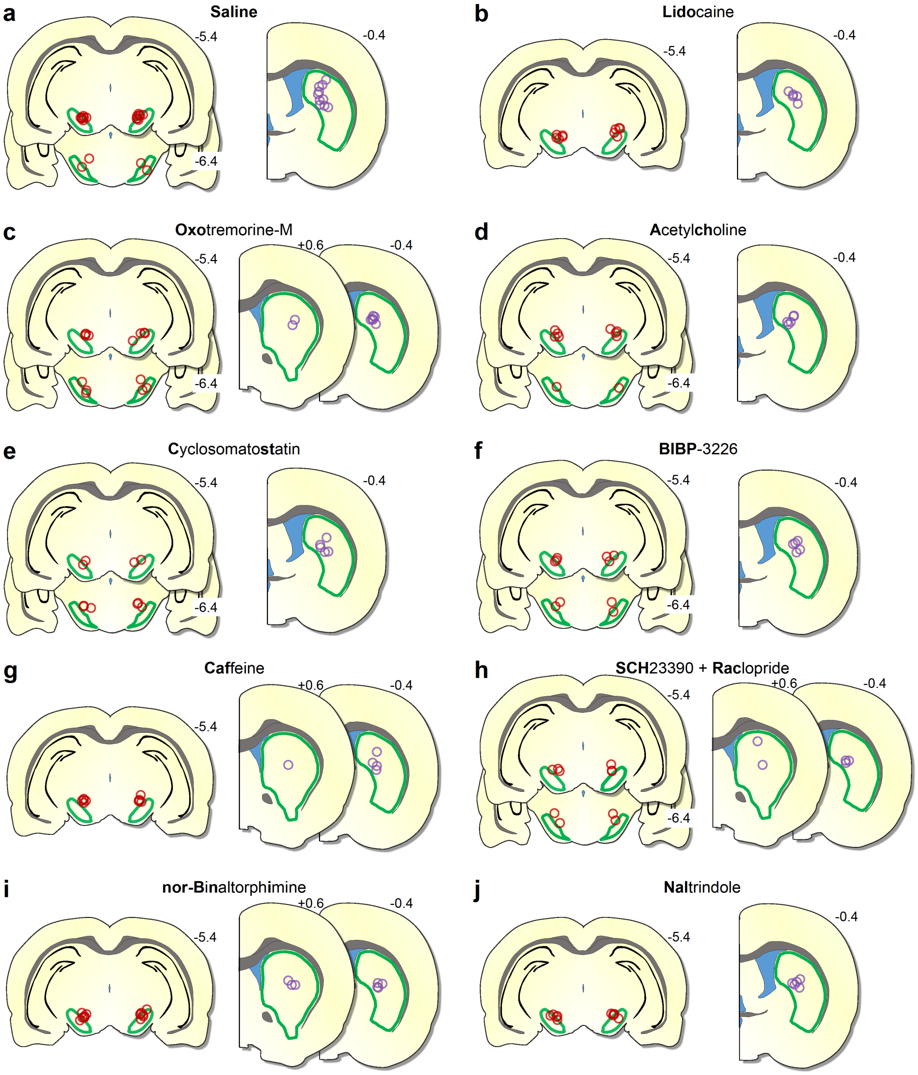

Fig. S8: Locations of stimulating optical fiber (red) and infusion cannula (purple) tips in subjects from pharmacological CBV-fMRI in Figure 4. a-j Anatomical boundaries for the optogenetic stimulation site (SNr) and drug infusion site (CPu) are outlined in green. The drug being infused is indicated at the top of each panel.

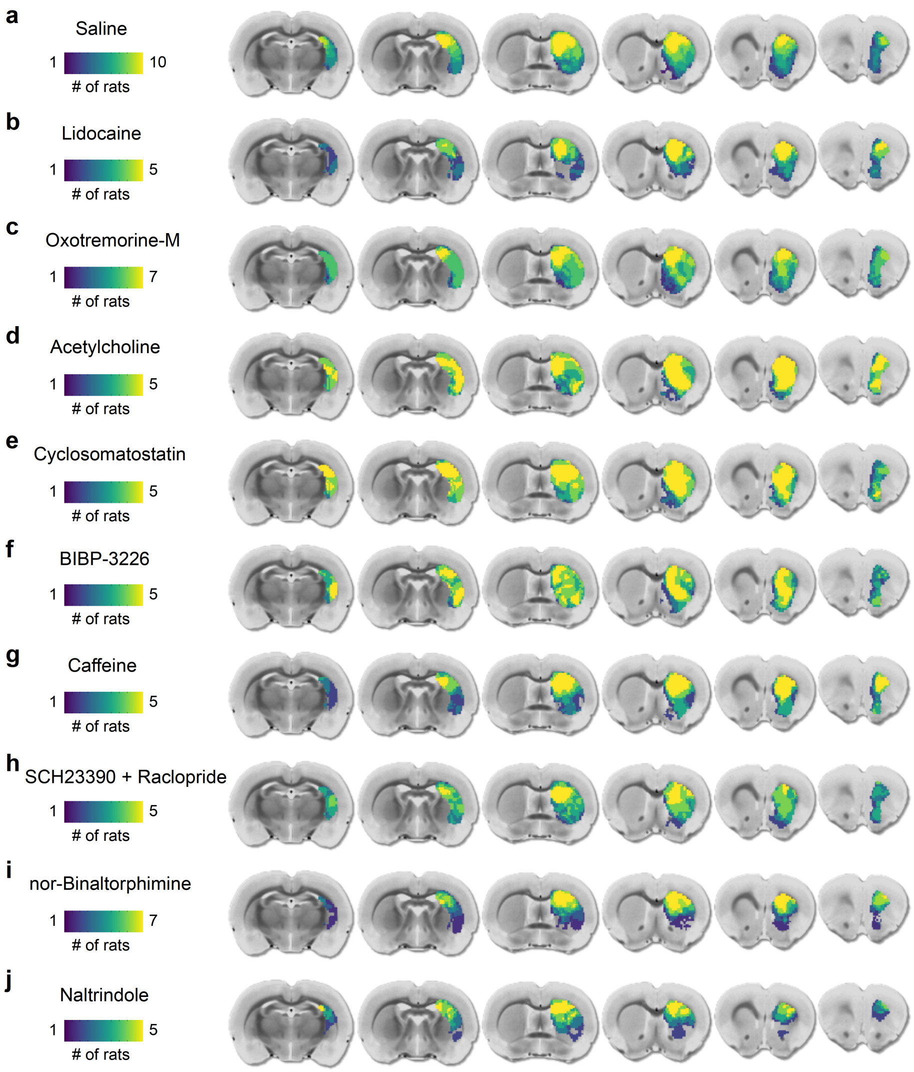

Fig. S9: Extended 6-slice ROI maps used for pharmacological CBV-fMRI studies in Figure 4. a-j ROIs used for timeseries extraction were determined by the intersection of individual subject pre-drug evoked response maps and the anatomical boundaries of the right CPu, extending from slices 2-7 out of 12, anterior to posterior. The drug being infused is indicated at the top left of each row.

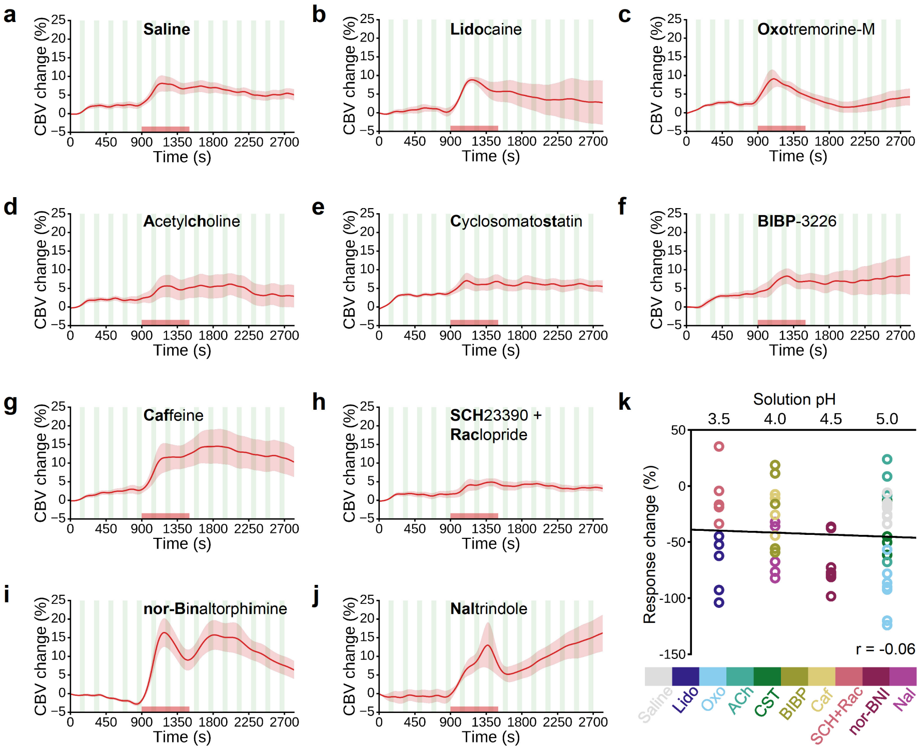

Fig. S10: Drug infusion controls from pharmacological CBV-fMRI experiments in Figure 4. a-j CBV baseline subtraction time-courses as a result of asymmetric least squares and quantile regression methods to correct for drug infusions (Time-course data are presented as mean ±SEM). The drug being infused is indicated at the top of each panel. Stimulation periods are indicated by green shaded bars and the infusion epochs are indicated by a shaded red bar on the x-axis. **k** Solution pH values obtained from freshly prepared drug in triplicate, compared to the percent change in CBV baseline during its in vivo infusion. Open circles indicate individual pH readings, color-coded to the appropriate drug name listed below. Source data are provided as a Source Data file.

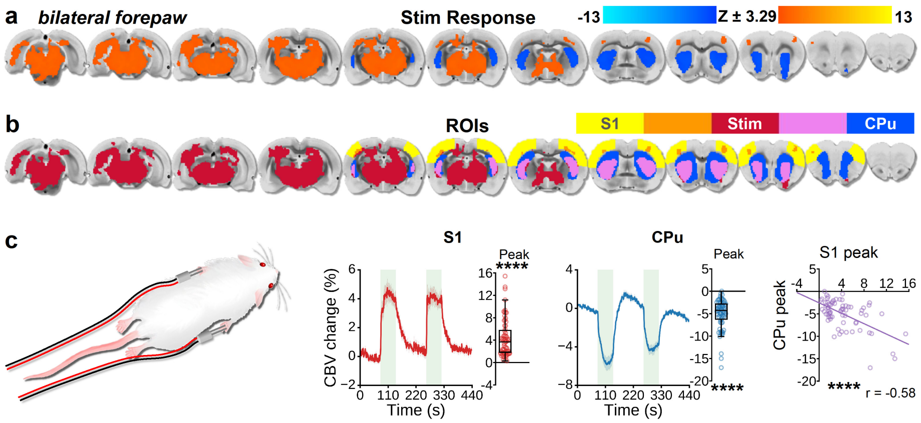

Fig. S11: Noxious forepaw stimulation elicits negative CBV-fMRI signals in rat CPu. Bilateral noxious electrical forepaw stimulation during CBV fMRI produced a significant positive bilateral response in the primary somatosensory cortex (S1) and a robust negative bilateral stimulus-evoked response in CPu (**a-c**), and these responses were linearly anticorrelated in peak amplitude (**c**, far right column). **a** Response maps acquired from subjects following bilateral noxious forepaw stimulation (n = 14 rats, 34 epochs)**.** Response maps thresholded to p<0.001 (two-tailed), FWE corrected to α < 0.01. **b** ROIs used for S1 and CPu timeseries extraction were collected from the intersection of the stimulus evoked response maps (red) and anatomical stimulation targets (yellow) or downstream anatomical areas of interest (blue), corresponding to the orange and purple regions, respectively. **a-b** Left-to-right corresponds to 1 mm steps in the posterior-to-anterior direction, with the 5th slice from the right located at the anterior commissure (approximately –0.36 mm AP). **c** Left to right columns: Bilateral forepaw stimulation schematic; CBV time-course from S1, aligned to stimulation epochs (n = 34 epochs; green bars indicate optogenetic stimulation blocks; data are presented as mean ±SEM), with corresponding quantified peak amplitude changes (n = 68 peaks; one-sample two-tailed t-test, ****p < 0.0001; box plots span IQR, with median line and whiskers within bounds ±1.5 IQR, using Tukey's method); corresponding CBV time-course and peak amplitude changes from CPu; Correlation plot between peak S1 and CPu CBV (linear regression two-tailed t-test, ****p < 0.0001). Exact p-values and test statistics are in Source Data. Source data are provided as a Source Data file.

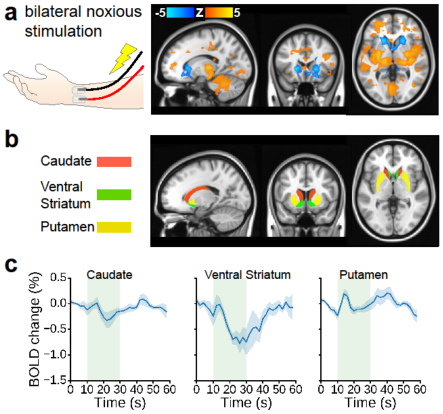

Fig. S12: Unmasked human response maps and striatum timeseries from noxious peripheral stimulation in Fig. 6b. a Unmasked human BOLD fMRI response maps corresponding to the masked response maps presented in Fig. 6b. **b** Human striatum anatomical ROI mask used for time-course extraction, color-coded by striatal compartment. **c** Stimulation aligned (green bars indicate stimulation ON), BOLD fMRI time-courses from each bilateral striatal compartment (n = 7 subjects, 11 scans; data are shown as subject mean ±SEM).

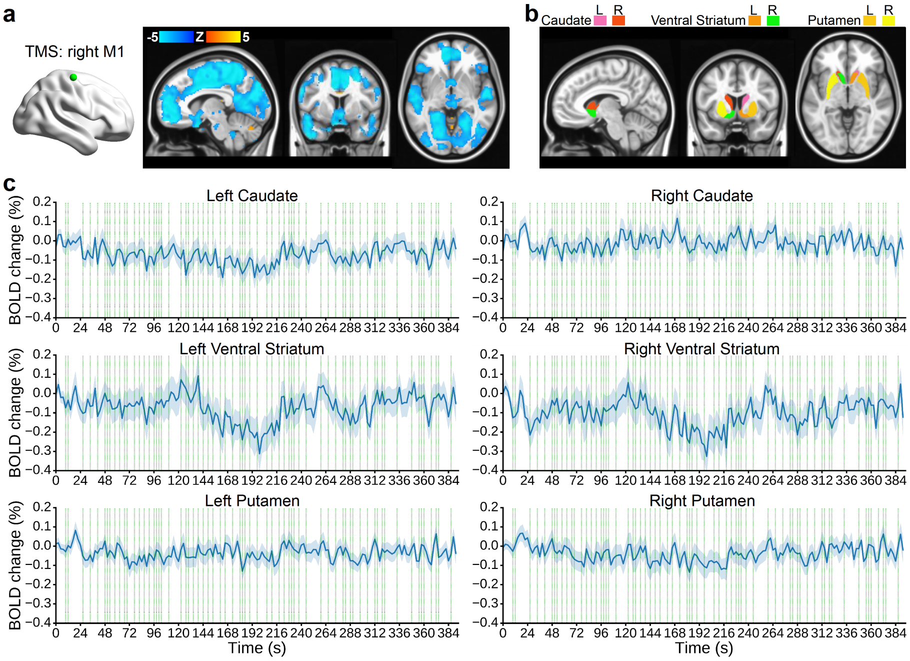

Fig. S13: Unmasked human response maps and striatum timeseries from right M1 TMS in Fig. 6c. a Unmasked human BOLD fMRI response maps corresponding to the masked response maps presented in Fig. 6c. **b** Human striatum anatomical ROI mask used for time-course extraction, color-coded by hemisphere and striatal compartment. **c** BOLD fMRI time-courses from each unilateral striatal compartment during fast event-related delivery of TMS pulses (pulse delivery was consistent between subjects and pulses are indicated by green bars; n = 79 subjects; data are shown as subject mean ±SEM).

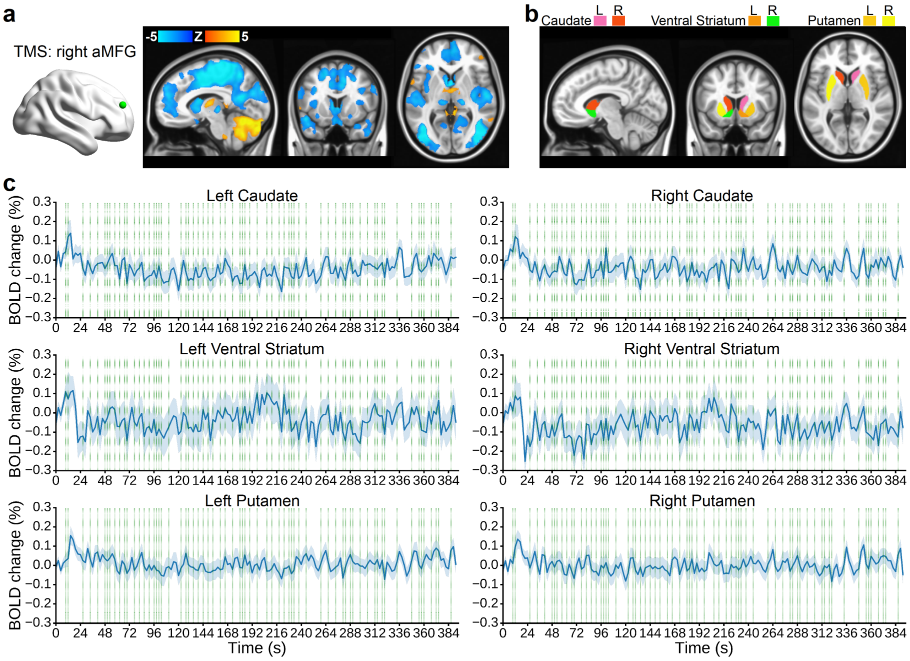

Fig. S14: Unmasked human response maps and striatum timeseries from right aMFG TMS in Fig. 6d. a Unmasked human BOLD fMRI response maps corresponding to the masked response maps presented in Fig. 6d. **b** Human striatum anatomical ROI mask used for time-course extraction, color-coded by hemisphere and striatal compartment. **c** BOLD fMRI time-courses from each unilateral striatal compartment during fast event-related delivery of TMS pulses (pulse delivery was consistent between subjects and pulses are indicated by green bars; n = 80 subjects; data are shown as subject mean ±SEM).

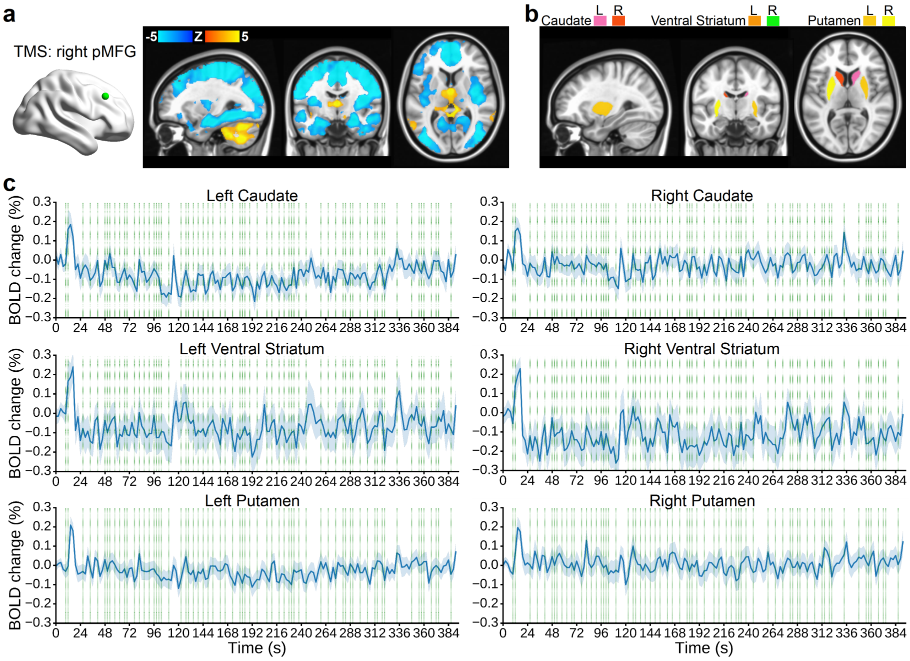

Fig. S15: Unmasked human response maps and striatum timeseries from right pMFG TMS in Fig. 6e. a Unmasked human BOLD fMRI response maps corresponding to the masked response maps presented in Fig. 6e. **b** Human striatum anatomical ROI mask used for time-course extraction, color-coded by hemisphere and striatal compartment. **c** BOLD fMRI time-courses from each unilateral striatal compartment during fast event-related delivery of TMS pulses (pulse delivery was consistent between subjects and pulses are indicated by green bars; n = 79 subjects; data are shown as subject mean ±SEM).

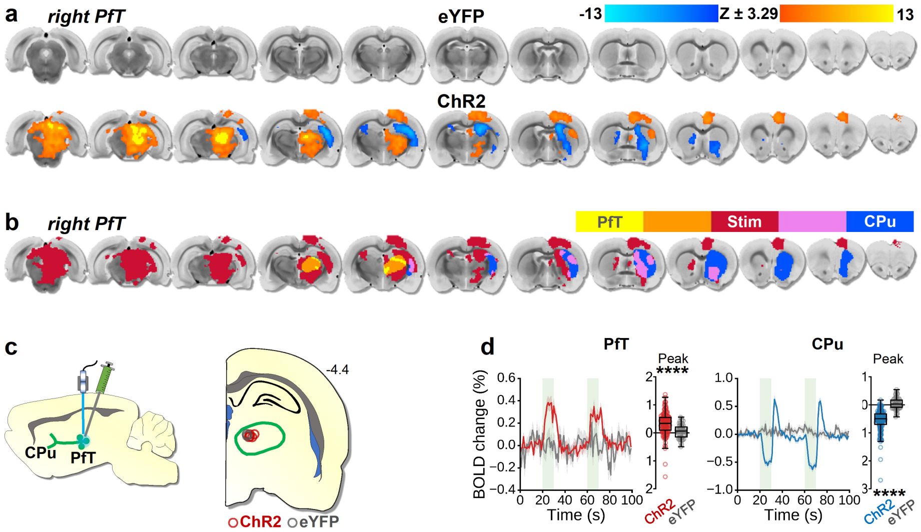

Fig. S16: Optogenetic stimulation of PfT during BOLD fMRI. a BOLD fMRI response maps acquired from eYFP control and ChR2 subjects following optogenetic stimulation of the right PfT. Response maps thresholded to $p < 0.001$ (two-tailed), FWE corrected to $\alpha < 0.01$. **b** ROIs used for timeseries extraction were collected from the intersection of the stimulus evoked response map (red) and anatomical boundary of PfT (yellow) or CPu (blue), corresponding to the orange and purple regions, respectively. **a-b** Left-to-right corresponds to 1 mm steps in the posterior-to-anterior direction, with the 5th slice from the right located at the anterior commissure (approximately –0.36 mm AP). **c** Stimulation schematics (left), where PfT viral expression and projections to CPu are indicated in green (not all projections shown) and optogenetically stimulated area of PfT is indicated in blue, and locations of optical fiber tips for PfT optogenetic stimulation (right). **d** BOLD time-courses from PfT response maps (left) and CPu response maps (right) aligned to stimulation epochs (green bars indicate 40 Hz optogenetic stimulation blocks; data are presented as mean ±SEM), with corresponding quantified peak amplitude changes (ChR2 vs. eYFP Welch's two-tailed t-test, ****$p < 0.0001$; box plots span IQR, with median line and whiskers within bounds ±1.5 IQR, using Tukey's method). **a-d** PfT stimulation during BOLD fMRI data (ChR2 $n = 5$ rats, 75 epochs, 150 peaks; eYFP $n = 2$ rats, 30 epochs, 60 peaks). While the time course extracted from the CPu ROI displayed negative BOLD changes during the stimulus period, we also observed a minor cluster exhibiting positive BOLD activation within the dorsolateral CPu. Notably, in a separate experiment employing identical manipulation, only negative CBV responses were elicited in the CPu (Fig. 1b, Fig. S2b). The increases in BOLD in dorsolateral CPu, potentially accompanied by decreases in CBV, could occur when CBV decreases substantially without prominent alterations in blood oxygenation, resulting in reduced deoxyhemoglobin occupancy and susceptibility effects. Alternatively, this observation may indeed be a result of the interplay between two competing forces within the CPu, as discussed in the main text of this manuscript: feed-forward vasoconstrictive neurotransmission versus

activity-driven metabolic processes. It is plausible that BOLD, being a contrast sensitive to multiple physiological factors, is capable of capturing these nuanced differences within distinct territories of the CPu that may not be discernible through CBV measurement alone.[3] Given the multifaceted nature of the BOLD signal, which can be influenced by varying alterations in blood oxygenation, blood flow, CBV, and local oxygen consumption, it is imperative that future multimodal neuroimaging studies are conducted to systematically dissect the underlying biophysical mechanisms driving this intriguing observation. Exact p-values and test statistics are in Source Data. Source data are provided as a Source Data file.

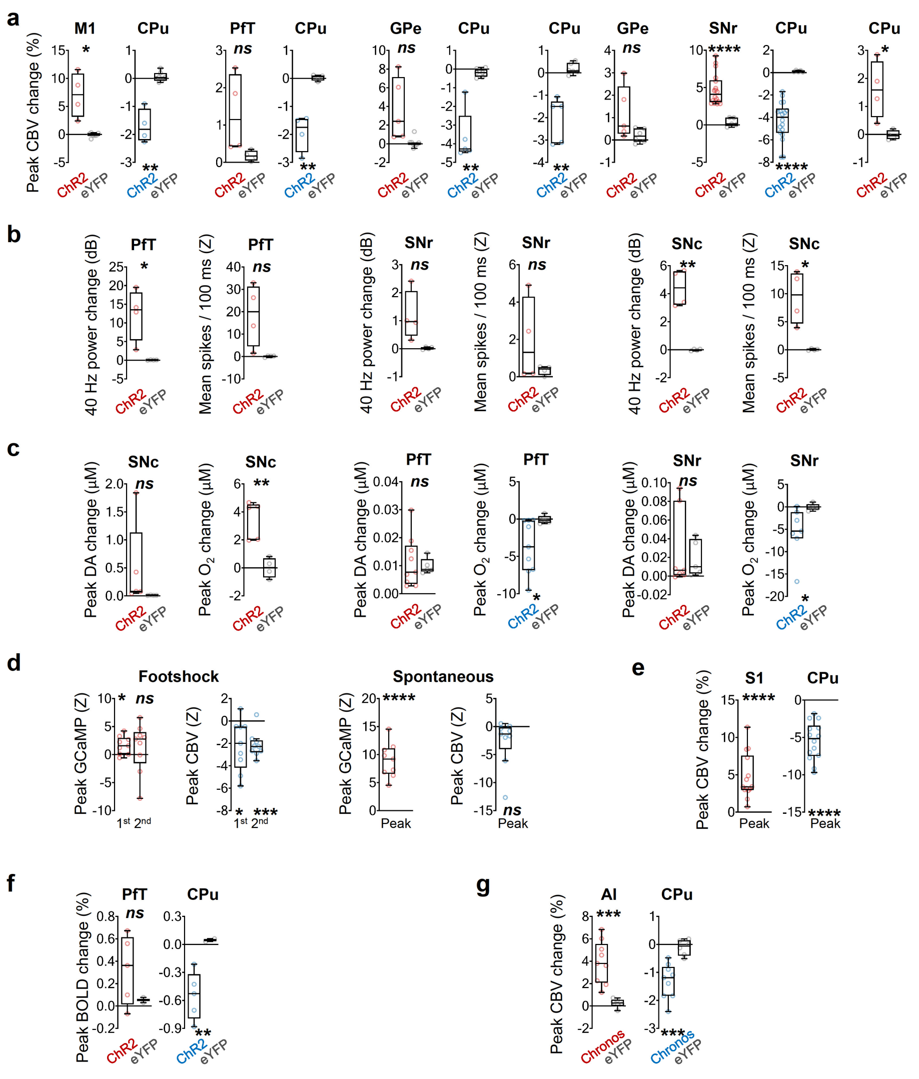

Fig. S17: Analyses reproductions with subject-level mean data. a (Fig. 1) CBV fMRI response peaks to optogenetic stimulation; from left to right: (Fig. 1a) M1 stimulation (ChR2 vs. eYFP two-tailed Welch's t-test, *$p < 0.05$, **$p < 0.01$); (Fig.

1b) PfT stimulation (ChR2 vs. eYFP two-tailed Welch's t-test, $^{ns}p > 0.05$, $^{**}p < 0.01$); (Fig. 1c) GPe stimulation (ChR2 vs. eYFP two-tailed Welch's t-test, $^{ns}p > 0.05$, $^{**}p < 0.01$); (Fig. 1d) CPu stimulation (ChR2 vs. eYFP two-tailed Welch's t-test, $^{ns}p > 0.05$, $^{**}p < 0.01$); (Fig. 1e) SNr stimulation (ChR2 vs. eYFP two-tailed Welch's t-test, $^{****}p < 0.0001$); (Fig. 1f) SNc stimulation (ChR2 vs. eYFP two-tailed Welch's t-test, $^{*}p < 0.05$). **b** (Fig. 2a-c) Electrophysiology 40Hz LFP power change and mean MUA spike frequency during optogenetic stimulation; from left to right: (Fig. 2a) PfT stimulation LFP (ChR2 vs. eYFP two-tailed Welch's t-test, $^{*}p < 0.05$) and MUA (ChR2 vs. eYFP two-tailed Welch's t-test, $^{ns}p > 0.05$); (Fig. 2b) SNr stimulation LFP (ChR2 vs. eYFP two-tailed Welch's t-test, $^{ns}p > 0.05$) and MUA (ChR2 vs. eYFP two-tailed Welch's t-test, $^{ns}p > 0.05$); (Fig. 2c) SNc stimulation LFP (ChR2 vs. eYFP two-tailed Welch's t-test, $^{**}p < 0.01$) and MUA (ChR2 vs. eYFP two-tailed Welch's t-test, $^{*}p < 0.05$). **c** (Fig. 2e-g) Voltammetry DA and O_2 response peaks to optogenetic stimulation; from left to right: (Fig. 2e) SNc stimulation DA (ChR2 vs. eYFP two-tailed Welch's t-test, $^{ns}p > 0.05$) and O_2 (ChR2 vs. eYFP two-tailed Welch's t-test, $^{**}p < 0.01$); (Fig. 2f) PfT stimulation DA (ChR2 vs. eYFP two-tailed Welch's t-test, $^{ns}p > 0.05$) and O_2 (ChR2 vs. eYFP two-tailed Welch's t-test, $^{*}p < 0.05$); (Fig. 2g) SNr stimulation DA (ChR2 vs. eYFP two-tailed Welch's t-test, $^{ns}p > 0.05$) and O_2 (ChR2 vs. eYFP two-tailed Welch's t-test, $^{*}p < 0.05$). **d** (Fig. 3) GCaMP and CBV photometry peaks from footshocks and relative to spontaneous GCaMP activity; from left to right: (fig. 3b) footshock response GCaMP (1st peak one-sample two-tailed t-test, $^{*}p < 0.05$; 2nd peak one-sample two-tailed t-test, $^{ns}p > 0.05$) and CBV (1st peak one-sample two-tailed t-test, $^{*}p < 0.05$; 2nd peak one-sample two-tailed t-test, $^{***}p < 0.0001$); (fig. 3c) spontaneous activity GCaMP (one-sample two-tailed t-test, $^{****}p < 0.0001$) and CBV (one-sample two-tailed t-test, $^{ns}p > 0.05$). **e** (Fig. S11) CBV fMRI response peaks to noxious forepaw stimulation (one-sample two-tailed t-test, $^{****}p < 0.0001$). **f** (Fig. S16) BOLD fMRI response peaks to optogenetic PfT stimulation (ChR2 vs. eYFP, two-tailed Welch's t-test, $^{ns}p > 0.05$, $^{**}p < 0.01$). **g** (Fig. S4) CBV fMRI response peaks to optogenetic AI stimulation (Chronos vs. eYFP two-tailed Welch's t-test, $^{***}p < 0.001$). Box plots span IQR, with median line and whiskers within bounds ±1.5 IQR, using Tukey's method. Exact p-values and test statistics are in Source Data. Source data are provided as a Source Data file.

References

1. Valdés-Hernández PA*, et al.* An in vivo MRI Template Set for Morphometry, Tissue Segmentation, and fMRI Localization in Rats. *Frontiers in neuroinformatics* **5**, 26 (2011).

2. Yushkevich PA*, et al.* User-guided 3D active contour segmentation of anatomical structures: Significantly improved efficiency and reliability. *NeuroImage* **31**, 1116-1128 (2006).

3. Drew PJ. Vascular and neural basis of the BOLD signal. *Curr Opin Neurobiol* **58**, 61-69 (2019).
