# Supplementary material for: Distinct neurochemical influences on fMRI response polarity in the striatum
Source: Nat Commun. 2024 Mar 1;15:1916. doi: 10.1038/s41467-024-46088-z (PMC10907631; doi:10.1038/s41467-024-46088-z)
Supplement: Supplementary file 3 — Reporting Summary [file 41467_2024_46088_MOESM3_ESM.pdf]

nature portfolio

Corresponding author(s): Yen-Yu Ian Shih

Last updated by author(s): Dec 17, 2023

Reporting Summary

Nature Portfolio wishes to improve the reproducibility of the work that we publish. This form provides structure for consistency and transparency in reporting. For further information on Nature Portfolio policies, see our Editorial Policies and the Editorial Policy Checklist.

Statistics

For all statistical analyses, confirm that the following items are present in the figure legend, table legend, main text, or Methods section.

n/a	Confirmed	
☐	☒	The exact sample size (*n*) for each experimental group/condition, given as a discrete number and unit of measurement
☐	☒	A statement on whether measurements were taken from distinct samples or whether the same sample was measured repeatedly
☐	☒	The statistical test(s) used AND whether they are one- or two-sided<br>*Only common tests should be described solely by name; describe more complex techniques in the Methods section.*
☐	☒	A description of all covariates tested
☐	☒	A description of any assumptions or corrections, such as tests of normality and adjustment for multiple comparisons
☐	☒	A full description of the statistical parameters including central tendency (e.g. means) or other basic estimates (e.g. regression coefficient) AND variation (e.g. standard deviation) or associated estimates of uncertainty (e.g. confidence intervals)
☐	☒	For null hypothesis testing, the test statistic (e.g. *F*, *t*, *r*) with confidence intervals, effect sizes, degrees of freedom and *P* value noted<br>*Give P values as exact values whenever suitable.*
☒	☐	For Bayesian analysis, information on the choice of priors and Markov chain Monte Carlo settings
☒	☐	For hierarchical and complex designs, identification of the appropriate level for tests and full reporting of outcomes
☐	☒	Estimates of effect sizes (e.g. Cohen's *d*, Pearson's *r*), indicating how they were calculated

Our web collection on statistics for biologists contains articles on many of the points above.

Software and code

Policy information about availability of computer code

Data collection

Rat MRI data was acquired using ParaVision 5 and ParaVision 6 preclinical imaging software (Bruker Corporation).

Electrophysiological data was acquired using Cerebus Central Suite (Ver.7.0.6).

Fast-scan cyclic voltammetry data was acquired with the High-Definition Cyclic Voltammetry software package (https://www.sciencedirect.com/science/article/pii/S1053811921009071#bib0008).

Fiber-photometry recordings were collected with the OceanView software package (Ver.1.5.).

All stimulus triggers for rat experiments were delivered according to the stimulation paradigms and synchronized to data acquisition via a DAQ board controlled by a homemade software program, PawStim.

Images of imunohistochemically prepared brain slices were obtained with the ZEN software package (Ver.3.4.).

Human MRI data was acquired with the Syngo MR Software (Ver.E11.) on the Siemens MAGNETOM scanner, and with the LX MR Software (Ver.12.) on the GE Signa Excite scanner.

Data analysis

MRI data was preprocessed, processed, and analyzed at the voxel-level with the open source Analysis of Functional NeuroImages (AFNI Ver.20.2.10) software suite.

Electrophysiological data was processed with the Neuroexplorer 5 software package.

Fast-scan cyclic voltammetry data was processed with the High-Definition Cyclic Voltammetry software package (https://www.sciencedirect.com/science/article/pii/S1053811921009071#bib0008).

Fiber-photometry data processing was performed in MATLAB (Ver.R2019b) using a published workflow (https://www.ncbi.nlm.nih.gov/pmc/articles/PMC9243291/).

Processing and analysis of confocal and bright-field imaged brain slices was performed in the open source FIJI ImageJ software package (Ver.1.50e.).

Human MRI TMS locations were defined with the Visor2 neuronavigation software and FMRIB Software Library (Ver.6.).

Customized data processing , analysis, and visualization scripts were executed in Python (Ver.2.7). The custom code used in this study has been archived on Zenodo: https://doi.org/10.5281/zenodo.7683340.

All other statistical tests and visualizations were generated in GraphPad Prism 9.

For manuscripts utilizing custom algorithms or software that are central to the research but not yet described in published literature, software must be made available to editors and reviewers. We strongly encourage code deposition in a community repository (e.g. GitHub). See the Nature Portfolio guidelines for submitting code & software for further information.

Data

Policy information about availability of data

All manuscripts must include a data availability statement. This statement should provide the following information, where applicable:

- Accession codes, unique identifiers, or web links for publicly available datasets
- A description of any restrictions on data availability
- For clinical datasets or third party data, please ensure that the statement adheres to our policy

Processed data used for discrete hypothesis testing, bar, box, and scatter plots, and the exact test statistics derived from these data are provided in the Source Data file.

All other data associated with this work, including those used for fMRI response maps, time-course plots, and peri-event spectrograms, are publicly available on Zenodo: https://doi.org/10.5281/zenodo.8417144.214

The human TMS fMRI data is publicly available on the NIMH data archives: https://nda.nih.gov/edit_collection.html?id=2856.

Source data are provided with this paper.

Human research participants

Policy information about studies involving human research participants and Sex and Gender in Research.

Reporting on sex and gender

Participants recruited for human fMRI experiments included both biologically male and female adults, as determined by self-reporting. Due to the limitation in sample size, sex- and gender-based analysis was not undertaken. The study did not aim to address sex differences, and such distinctions are outside its scope. Similar to the rodent counterpart of this study, sex differences were not incorporated as part of the hypothesis. The manuscript addresses this limitation and discusses potential sex-dependent findings.

Population characteristics

Seven healthy right-handed subjects (6 males and 1 female, 32.1 ± 5.5 years old, mean ± SD) participated in the TENS fMRI experiment. None of the subjects had any form of acute or chronic pain, or took drugs that affect pain sensations or the central nervous system.

For each stimulation site in the TMS fMRI experiment, the population characteristics of the participants are as follows: aMFG, 46 females, 34 males, 31.9(10.7 s.d.) years old, 16.2(2.1 s.d.) years of education; pMFG, 47 females, 32 males, 31.7(10.7 s.d.) years old, 16.1 (2.1 s.d.) years of education; M1, 48 females, 31 males, 31.7(10.6 s.d.) years old, 16.2(2.1 s.d.) years of education.

Recruitment

TENS fMRI participants were recruited from members of the Center for Animal MRI at the University of North Carolina at Chapel Hill for study efficiency. While it is unlikely for these participants exhibit distinct striatal fMRI responses, this recruitment approach might introduce a selection bias to the study. TMS fMRI participants were recruited through community advertisements at the Stanford Medical School.

Ethics oversight

The TENS fMRI experiment was approved by the Institutional Review Board at the University of North Carolina at Chapel Hill.

The TMS fMRI experiment was approved by the Stanford Institutional Review Board (Protocol #25948).

Note that full information on the approval of the study protocol must also be provided in the manuscript.

Field-specific reporting

Please select the one below that is the best fit for your research. If you are not sure, read the appropriate sections before making your selection.

☒ Life sciences ☐ Behavioural & social sciences ☐ Ecological, evolutionary & environmental sciences

For a reference copy of the document with all sections, see nature.com/documents/nr-reporting-summary-flat.pdf

Life sciences study design

All studies must disclose on these points even when the disclosure is negative.

Sample size	No sample size calculations were performed. Sample sizes for individual experiments were chosen based on our previously published studies with rat optogenetic-fMRI experiments ((https://doi.org/10.1038/srep31613 , https://doi.org/10.1016%2Fj.neuroimage.2016.02.067) and are consistent with those (not considering group distribution) used for rat fMRI experiments as a whole (https://doi.org/10.3389/fninf.2019.00078). To ensure statistical rigor, we used a minimum of n = 4 subjects for all in vivo experiments to account for subject-level variability. Sample size was determined to be sufficient based on the magnitude and consistency of measurable differences between groups and experimental conditions.
Data exclusions	No data were excluded from the analyses.
Replication	Although the experiments reported here show consistent results between dependent and independent measurements, we did not explicitly replicate experiments to minimize costs, equipment usage, and animal numbers. Nonetheless, our main findings are corroborated between experiments (e.g., negative hemodynamic signals in CPu to stimulation were observed across three modalities and in two species, etc.). To promote reproducibility, the data used in this study has been made publicly available between Zenodo (https://doi.org/10.5281/zenodo.8417144.214) and the Source Data file, and custom code used in this study has been made publicly available on Zenodo: https://doi.org/10.5281/zenodo.7683340.
Randomization	Subjects were randomly assigned to conditions within an experiment or control groups at the time of surgical preparation.
Blinding	Investigators were not blinded to group allocation for fMRI, electrophysiology, voltammetry, and fiber-photometry experiments, however data analysis was performed automatically and with software scripts and not subject to experimenter bias. Analysis of anatomical MRI, confocal microscopy, and bright-field microscopy data was performed by technicians not involved in sample preparation/data acquisition and blinded to the experimental design.

Reporting for specific materials, systems and methods

We require information from authors about some types of materials, experimental systems and methods used in many studies. Here, indicate whether each material, system or method listed is relevant to your study. If you are not sure if a list item applies to your research, read the appropriate section before selecting a response.

Materials & experimental systems

n/a	Involved in the study	
☐	☒	Antibodies
☒	☐	Eukaryotic cell lines
☒	☐	Palaeontology and archaeology
☐	☒	Animals and other organisms
☒	☐	Clinical data
☒	☐	Dual use research of concern

Methods

n/a	Involved in the study	
☒	☐	ChIP-seq
☒	☐	Flow cytometry
☐	☒	MRI-based neuroimaging

Antibodies

Antibodies used

Primary:
DARPP-32 (#611520, BD Biosciences, Haryana, India)
ChAT (#50-265, ProSci, Poway, CA)
PV (ab11427, Abcam, Cambridge, UK)
TH (AB152, MilliporeSigma, Burlington, MA)

Secondary:
EnVision+ System- HRP, labelled polymer, anti-mouse (K4001, Dako, Santa Clara, CA)
ImmPRESS HRP anti-goat IG (MP-7405, VectorLabs, Newark, CA)
EnVision+ System- HRP, labelled polymer, anti-rabbit (K4003, Dako, Santa Clara, CA)
Goat anti-rabbit conjugated to Alexa568 (A11036, Invitrogen, Waltham, MA)

Validation	The Technical Data Sheet for the DARPP-32 antibody can be found at: https://www.bdbiosciences.com/content/bdb/paths/generate-tds-document.us.611520.pdf . This antibody was validated by western blot and immunofluorescence, and appears in the following citations:<br>Bibb JA, Snyder GL, Nishi A, Yan Z. Phosphorylation of DARPP-32 by Cdk5 modulates dopamine signalling in neurons. Nature. 1999; 402(6762):669-671. (Biology).<br>Fienberg AA, Hiroi N, Mermelstein PG. DARPP-32: regulator of the efficacy of dopaminergic neurotransmission. Science. 1998; 281(5378):838-842. (Biology).<br>Kurihara T, Lewis RM, Esler J, Greengard P. Cloning of cDNA for DARPP-32, a dopamine- and cyclic AMP-regulated neuronal phosphoprotein. J Neurosci. 1988; 8(2):508-517. (Biology).<br>Yan Z, Feng J, Fienberg AA, Greengard P. D(2) dopamine receptors induce mitogen-activated protein kinase and cAMP response element-binding protein phosphorylation in neurons. Proc Natl Acad Sci U S A. 1999; 96(20):11607-11612. (Biology).<br>Yan Z, Hsieh-Wilson L, Feng J. Protein phosphatase 1 modulation of neostriatal AMPA channels: regulation by DARPP-32 and spinophilin. Nat Neurosci. 1999; 2(1):13-17. (Biology).<br><br>The Technical Data Sheet for the ChAT antibody can be fund at: https://www.prosci-inc.com/wp-content/uploads/product-datasheet-pdfs/choline-acetyltransferase-antibody-50-265.datasheet.pdf . This antibody was validated by immunohistochemistry and western blot, and appears in the following citations:<br>Galvan A, Hu X, Smith Y, Wichmann T. In vivo optogenetic control of striatal and thalamic neurons in non-human primates. PLoS One. 2012;7(11):e50808. doi: 10.1371/journal.pone.0050808. Epub 2012 Nov 30. PMID: 23226390; PMCID: PMC3511281.<br>Lallani SB, Villalba RM, Chen Y, Smith Y, Chan AWS. Striatal Interneurons in Transgenic Nonhuman Primate Model of Huntington's Disease. Scientific Reports. 2019 Mar;9(1):3528. DOI: 10.1038/s41598-019-40165-w. PMID: 30837611; PMCID: PMC6401084.<br>Yalcin-Cakmakli G, Rose SJ, Villalba RM, Williams L, Jinnah HA, Hess EJ, Smith Y. Striatal Cholinergic Interneurons in a Knock-in Mouse Model of L-DOPA-Responsive Dystonia. Front Syst Neurosci. 2018 Jun 27;12:28. doi: 10.3389/fnsys.2018.00028. PMID: 29997483; PMCID: PMC6030733.<br><br>The Technical Data Sheet for the PV antibody can be found at: https://www.abcam.com/products/primary-antibodies/parvalbumin-antibody-ab11427.pdf . This antibody was validated by Immunocytochemistry/Immunofluorescence and Immunohistochemistry, and appears in the following citations:<br>Fredes F, Silva MA, Koppensteiner P, Kobayashi K, Joesch M, Shigemoto R. Ventro-dorsal Hippocampal Pathway Gates Novelty-Induced Contextual Memory Formation. Curr Biol. 2021 Jan 11;31(1):25-38.e5. doi: 10.1016/j.cub.2020.09.074. Epub 2020 Oct 15. PMID: 33065009; PMCID: PMC7808756.<br>Biagioni F, Vivacqua G, Lazzeri G, Ferese R, Iannacone S, Onori P, Morini S, D'Este L, Fornai F. Chronic MPTP in Mice Damage-specific Neuronal Phenotypes within Dorsal Laminae of the Spinal Cord. Neurotox Res. 2021 Apr;39(2):156-169. doi: 10.1007/s12640-020-00313-x. Epub 2020 Nov 18. PMID: 33206341; PMCID: PMC7936970.<br>Briones BA, Pisano TJ, Pitcher MN, Haye AE, Diethorn EJ, Engel EA, Cameron HA, Gould E. Adult-born granule cell mossy fibers preferentially target parvalbumin-positive interneurons surrounded by perineuronal nets. Hippocampus. 2021 Apr;31(4):375-388. doi: 10.1002/hipo.23296. Epub 2021 Jan 12. PMID: 33432721; PMCID: PMC8020456.<br><br>The description of the TH antibody can be found at: https://www.sigmaaldrich.com/US/en/product/mm/ab152 . This antibody was validated by ELISA, immunofluorescence, immunohistochemistry, western blot, and immuniprecipitation, and appears in the following citations:<br>Plaisier F, Hume C, Menzies J. Neural connectivity between the hypothalamic supramammillary nucleus and appetite- and motivation-related regions of the rat brain. J Neuroendocrinol. 2020 Feb;32(2):e12829. doi: 10.1111/jne.12829. Epub 2020 Jan 29. PMID: 31925973; PMCID: PMC7065010.<br>Kempadoo KA, Mosharov EV, Choi SJ, Sulzer D, Kandel ER. Dopamine release from the locus coeruleus to the dorsal hippocampus promotes spatial learning and memory. Proc Natl Acad Sci U S A. 2016 Dec 20;113(51):14835-14840. doi: 10.1073/pnas.1616515114. Epub 2016 Dec 7. PMID: 27930324; PMCID: PMC5187750.<br>Orlando R, Ginerete RP, Cavalleri L, Aliperti V, Imbriglio T, Battaglia G, Zuena AR, Nicoletti F, Merlo Pich E, Collo G. Synergic action of L-acetylcarnitine and L-methylfolate in Mouse Models of Stress-Related Disorders and Human iPSC-Derived Dopaminergic Neurons. Front Pharmacol. 2022 Jun 2;13:913210. doi: 10.3389/fphar.2022.913210. PMID: 35721218; PMCID: PMC9201783.

Animals and other research organisms

Policy information about studies involving animals; ARRIVE guidelines recommended for reporting animal research, and Sex and Gender in Research

Laboratory animals	Sprague-Dawley rats (male, n = 139, 350-600 g, postnatal day 80-300) were obtained from Charles River (Wilmington, MA, USA)<br><br>TH-Cre transgenic Long-Evans rats (male, n = 28; 400-600 g, postnatal day 120-300) were acquired from the Rat Resource and Research Center (P40OD011062; Columbia, MO, USA)<br><br>Wistar rats (n = 4, ~350 g, 3 males and 1 female, postnatal day 80-200), born and reared at the University of North Carolina-Chapel Hill, were used for an orbitofrontal cortex (OFC) optogenetic fMRI study shown in the supplementary information.<br><br>C57BL/6J mice (n = 3, 1 male, postnatal day 21--28) were bred in house at the University of Sussex.
Wild animals	This study did not involve wild animals.
Reporting on sex	Sex was not considered in the design of this study. Except for one subject in the OFC experiment cohort, all rats used herein were identified as male by the supplier. Mice were identified as both sexes by technicians at the University of Sussex. While both sexes are recruited in the studies, the sample size is insufficient to investigate sex-related differences.
Field-collected samples	This study did not involve samples collected from the field.

Ethics oversight

Rat care and handling followed the National Institutes of Health Guide for the Care and Use of Laboratory Animals (Department of Health and Human Services, NIH publication No. 86-23, revised 1985), and all rat protocols were approved by the Institutional Animal Care and Use Committee at UNC (Protocol # 16-296 and 15-057).

Mouse procedures were carried out in accordance with the guidelines of the UK Animals (Scientific Procedures) Act 1986, the Danish National Ethics Committee and European Directive 2010/63/EU.

Note that full information on the approval of the study protocol must also be provided in the manuscript.

Magnetic resonance imaging

Experimental design

Design type

Rat fMRI and Human TENS fMRI were block designs. Human TMS fMRI was an event-related design.

Design specifications

Rat fMRI optogenetic and electrical stimulation trials included 2 pulsed stimulation "ON" blocks separated by a stimulation "OFF" block. Trials were separated by the duration of the OFF block at minimum. Pharmacological fMRI trials with SNr stimulation included 15 ON blocks separated by OFF blocks, with a 600 s intracranial drug infusion starting after the 5th ON block.The duration of ON and OFF blocks for each stimulation target were as follows:
M1 - 10 s ON, 30 s OFF
PfT - 10 s ON, 30 s OFF
GPe - 10 s ON, 30 s OFF
CPu - 10 s ON, 30 s OFF
SNr - 60 s ON, 120 s OFF
SNc - 10 s ON, 30 s OFF
AI - 20 s ON, 80 s OFF
OFC - 15 s ON, 60 s OFF
Forepaw - 60 s ON, 120 s OFF

Human TENS fMRI trials included 6 20 s ON blocks flanked by 30 s OFF blocks.

Human TMS trials included 68 TMS pulses presented with a variable inter-stimulus interval jittered with delays of 2.4, 4.2, and 7.2 s delivered over 6 min and 41 s.

Behavioral performance measures

This study did not involve behavioral performance measures.

Acquisition

Imaging type(s)

Functional and structural/anatomical.

Field strength

Rat acquisitions were at 9.4-Tesla. Human TENS acquisitions were at 7-Tesla. Human TMS acquisitions were at 1.5-Tesla.

Sequence & imaging parameters

Rat anatomical MRI scans were acquired with the following parameters:
Sequence = T2-weighted RARE, spectral width = 47 kHz, TR/TE = 2500/33 ms, FOV = 2.56x2.56 mm2 , matrix size = 256x256, RARE factor = 8, averages = 8.

Rat CBV fMRI scans were acquired with the following parameters:
Sequence = single shot, gradient echo EPI sequence, spectral width = 300 kHz, TR/TE = 1000/8 ms, FOV = 2.56 x 2.56 cm2, matrix size = 80x80.

Human anatomical MRI scans for TENS experiments were acquired with the following parameters:
Sequence = T1-weighted, TR/TE = 2200/2.86 ms, in-plane matrix 256 x 248, 0.859 x 0.859 x 1 mm3.

Human TENS fMRI scans were acquired with the following parameters:
Sequence = gradient EPI, TR/TE = 2000/25 ms, flip angle 926 = 79°, in-plane matrix 74 x 72, 2.973 x 2.973 x 3 mm3.

In human TMS MRI experiments, a high resolution T1-weighted anatomical image collected at a separate scanning session was used to define TMS stimulation sites for each participants.

Human TMS fMRI scans were acquired with the following parameters:
Sequence = T2-weighted gradient echo spiral in/out pulse sequence, TR = 2400 ms, TE = 30 ms, flip angle = 85°, 1 interleave, FOV = 22 cm, pixel size = 3.4 mm, 64 x 64 matrix.

Area of acquisition

In general, whole brain scans were used. However, rat MRI images did not include the anterior- and posterior-most portions of the brain. Specifically, rat MRI images were acquired in anisotropic resolution with 1 mm thick coronal slices spaced at 1 mm intervals and aligned so that the fifth slice from the front of each animal's head was aligned to the anterior commissure (AC) in the mid-sagittal plane (0.36 mm AP relative to bregma); M1 optogenetic stimulation scans were 8 slices with the 4th from the front corresponding to the AC.

Diffusion MRI ☐ Used ☒ Not used

Preprocessing

Preprocessing software	MRI data was preprocessed, processed, and analyzed at the voxel-level with the open source Analysis of Functional NeuroImages (AFNI Ver.20.2.10) software suite. For each fMRI experiment, images were motion-corrected, slice-timing corrected, and coregistered to template space. Functional images were also smoothed with a gaussian kernal (0.5 mm FWHM for rat data, 4 mm for human data). In addition, rat fMRI images were skull-stripped with hand-drawn brain masks prior to template coregistration.
Normalization	Rat fMRI data was normalized by first linearly coregistering functional to corresponding anatomical image data, then linearly coregistering anatomical images to Tohoku template space, and finally applying the anatomical-to-template affine transformation matrix to the functional images.<br><br>Human fMRI data was normalized by first linearly coregistering the anatomical images from each subject to MNI-152 template space, then linearly coregistering functional to corresponding anatomical image data, and finally linearly coregistering the functional images to MNI-152 template space.
Normalization template	Rat MRI data was normalized to the Tohoku rat brain template space and human MRI data was normalized to MNI-152 template space.
Noise and artifact removal	Nuisance removal included motion correction via regressing out head motion parameters.
Volume censoring	Volume censoring was not used in this study.

Statistical modeling & inference

Model type and settings	We used a conventional general linear model (3dDeconvolve) as implemented in AFNI to determine subject-level brain activation maps. Coefficients were determined for voxel-wise timeseries data against the stimulation block/event design convoluted with the AFNI "BLOCK" function for rat fMRI data, and "BOLD" function for human fMRI data. In addition, coefficients for rat fMRI data were calculated with the AFNI REMLfit package, using linear mixed-effects modeling (on the aforementioned factors) with restricted maximum likelihood (REML) estimates applied to correct for temporal autocorrelations.
Effect(s) tested	Separate one sample t-tests (AFNI 3dttest++) were used to determine group-level activation maps to each experimental stimulation paradigm.

Specify type of analysis: ☐ Whole brain ☐ ROI-based ☒ Both

Anatomical location(s)	ROIs for rat fMRI timeseries extraction were generated using the intersection of group-level stimulation-response maps and hand-drawn anatomical areas of the target regions according to the Paxinos and Watson 6th edition rat brain atlas, coregistered to template space.<br><br>General linear model analysis of human fMRI data was restricted to the striatum using a mask corresponding to the anatomical boundaries of striatum in MNI-152 space.

Statistic type for inference (See Eklund et al. 2016)	Rat fMRI response maps were thresholded to $p = 0.001$, then corrected for family-wise error to adjust for multiple comparisons using bi-sided cluster-size thresholds ($\alpha < 0.01$) determined automatically for each comparison by the 3dClustSim AFNI program.<br><br>Voxel-wise statistics were used for inferences on human fMRI data, with thresholding at $p < 0.05$ after accounting for multiple comparisons.
Correction	The FWE cluster-correction threshold for rat fMRI data was automatically determined at ($\alpha < 0.01$) for each comparison by the 3dClustSim AFNI program.<br><br>For human fMRI data, the false discovery rate correction was used to adjust for multiple comparisons of the fMRI maps ($p < 0.05$).

Models & analysis

n/a	Involved in the study
☒	☐ Functional and/or effective connectivity
☒	☐ Graph analysis
☒	☐ Multivariate modeling or predictive analysis
